# Supplementary material for: Characterizing cell-type spatial relationships across length scales in spatially resolved omics data
Source: Nat Commun. 2025 Jan 3;16:350. doi: 10.1038/s41467-024-55700-1 (PMC11699133; doi:10.1038/s41467-024-55700-1)
Supplement: Supplementary file 3 — Supplementary Software 1 [file 41467_2024_55700_MOESM3_ESM.zip › CRAWDAD/plots/supplementary/S7_cluster_corr_heatmap_PKHL_vs_KSFB.pdf]

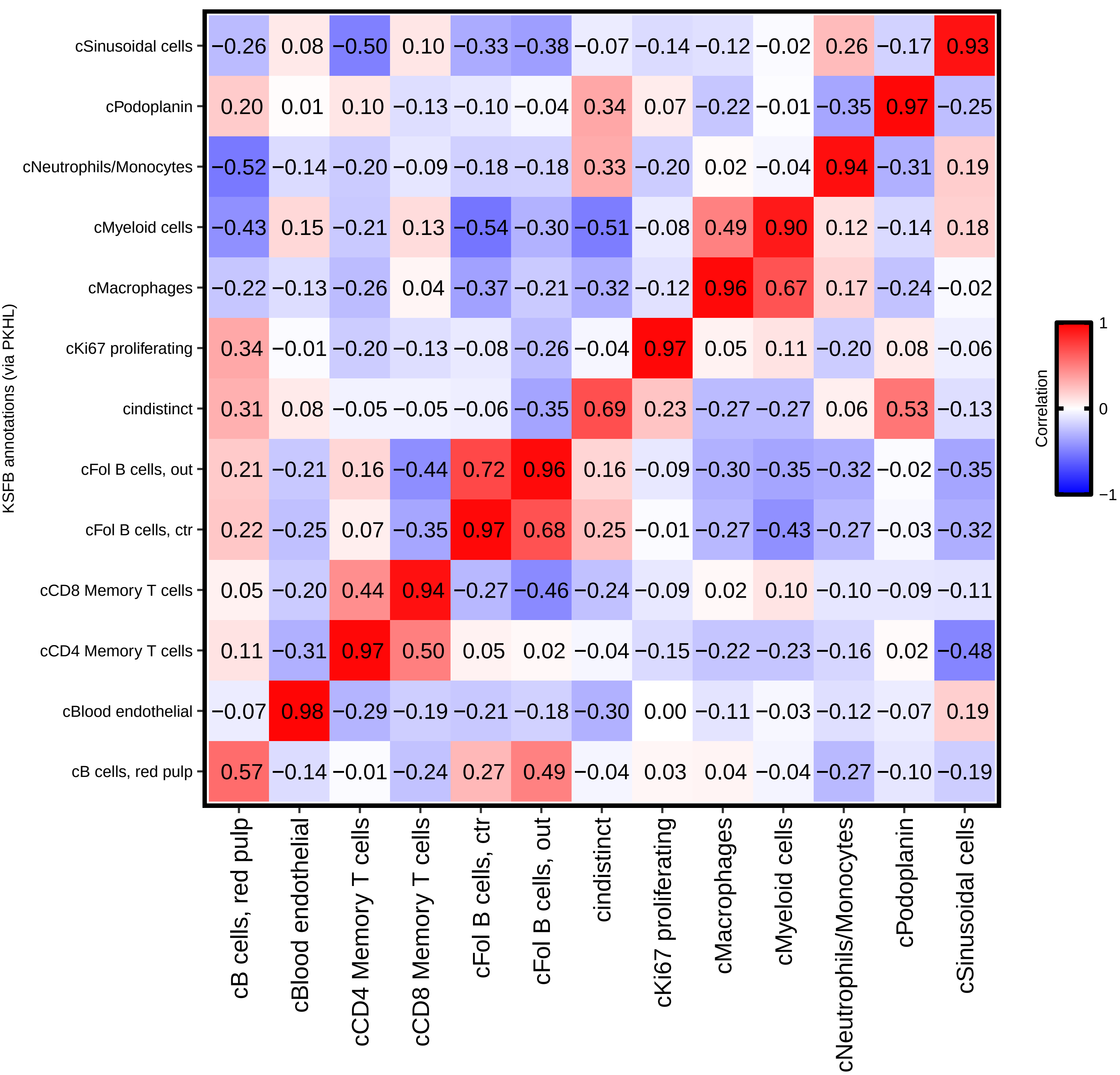

cSinusoidal cells
cPodoplanin
cNeutrophils/Monocytes
cMyeloid cells
cMacrophages
cKi67 proliferating
cindistinct
cFol B cells, out
cFol B cells, ctr
cCD8 Memory T cells
cCD4 Memory T cells
cBlood endothelial
cB cells, red pulp
−0.26 0.08 −0.50 0.10 −0.33 −0.38 −0.07 −0.14 −0.12 −0.02 0.26 −0.17 0.93
0.20 0.01 0.10 −0.13 −0.10 −0.04 0.34 0.07 −0.22 −0.01 −0.35 0.97 −0.25
−0.52 −0.14 −0.20 −0.09 −0.18 −0.18 0.33 −0.20 0.02 −0.04 0.94 −0.31 0.19
−0.43 0.15 −0.21 0.13 −0.54 −0.30 −0.51 −0.08 0.49 0.90 0.12 −0.14 0.18
−0.22 −0.13 −0.26 0.04 −0.37 −0.21 −0.32 −0.12 0.96 0.67 0.17 −0.24 −0.02
0.34 −0.01 −0.20 −0.13 −0.08 −0.26 −0.04 0.97 0.05 0.11 −0.20 0.08 −0.06
0.31 0.08 −0.05 −0.05 −0.06 −0.35 0.69 0.23 −0.27 −0.27 0.06 0.53 −0.13
0.21 −0.21 0.16 −0.44 0.72 0.96 0.16 −0.09 −0.30 −0.35 −0.32 −0.02 −0.35
0.22 −0.25 0.07 −0.35 0.97 0.68 0.25 −0.01 −0.27 −0.43 −0.27 −0.03 −0.32
0.05 −0.20 0.44 0.94 −0.27 −0.46 −0.24 −0.09 0.02 0.10 −0.10 −0.09 −0.11
0.11 −0.31 0.97 0.50 0.05 0.02 −0.04 −0.15 −0.22 −0.23 −0.16 0.02 −0.48
−0.07 0.98 −0.29 −0.19 −0.21 −0.18 −0.30 0.00 −0.11 −0.03 −0.12 −0.07 0.19
0.57 −0.14 −0.01 −0.24 0.27 0.49 −0.04 0.03 0.04 −0.04 −0.27 −0.10 −0.19
KSFB annotations (via PKHL)
cB cells, red pulp
cBlood endothelial
cCD4 Memory T cells
cCD8 Memory T cells
cFol B cells, ctr
cFol B cells, out
cindistinct
cKi67 proliferating
cMacrophages
cMyeloid cells
cNeutrophils/Monocytes
cPodoplanin
cSinusoidal cells
PKHL
Correlation
1
0
−1
